# Supplementary material for: Modeling glioblastoma heterogeneity as a dynamic network of cell states
Source: Mol Syst Biol. 2021 Sep 16;17(9):e10105. doi: 10.15252/msb.202010105 (PMC8444284; doi:10.15252/msb.202010105)
Supplement: Supplementary file 5 — Source Data for Figure 3 [file MSB-17-e10105-s001.zip › Figure3A_sourcedata/GSEA_3065/nosigdb_state4.GseaPreranked.1622718430994/POLLEN_OUTERRADIALGLIA_PMID_26406371.html]

Details for gene set POLLEN\_OUTERRADIALGLIA\_PMID\_26406371[GSEA]

|  || Dataset | state4 |
| Phenotype | NoPhenotypeAvailable |
| Upregulated in class | na\_pos |
| GeneSet | POLLEN\_OUTERRADIALGLIA\_PMID\_26406371 |
| Enrichment Score (ES) | 0.5728741 |
| Normalized Enrichment Score (NES) | 1.9635383 |
| Nominal p-value | 0.0 |
| FDR q-value | 0.0020777865 |
| FWER p-Value | 0.015 |
Table: GSEA Results Summary

  

Fig 1: Enrichment plot: POLLEN\_OUTERRADIALGLIA\_PMID\_26406371      
 Profile of the Running ES Score & Positions of GeneSet Members on the Rank Ordered List

  

| PROBE | GENE SYMBOL | GENE\_TITLE | RANK IN GENE LIST | RANK METRIC SCORE | RUNNING ES | CORE ENRICHMENT || 1 | GFAP |  |  | 0 | 0.995 | 0.1661 | Yes |
| 2 | SAT1 |  |  | 4 | 0.669 | 0.2773 | Yes |
| 3 | FABP7 |  |  | 5 | 0.656 | 0.3868 | Yes |
| 4 | HOPX |  |  | 28 | 0.360 | 0.4433 | Yes |
| 5 | MT3 |  |  | 38 | 0.318 | 0.4949 | Yes |
| 6 | LGALS3 |  |  | 89 | 0.259 | 0.5301 | Yes |
| 7 | TTYH1 |  |  | 100 | 0.249 | 0.5700 | Yes |
| 8 | EDNRB |  |  | 271 | 0.180 | 0.5729 | Yes |
| 9 | ABAT |  |  | 435 | 0.145 | 0.5710 | No |
| 10 | MLC1 |  |  | 594 | 0.126 | 0.5666 | No |
| 11 | PMP22 |  |  | 765 | 0.107 | 0.5571 | No |
| 12 | SEMA5A |  |  | 1036 | 0.085 | 0.5280 | No |
| 13 | ETV1 |  |  | 1199 | 0.076 | 0.5147 | No |
| 14 | TFPI |  |  | 1213 | 0.075 | 0.5252 | No |
| 15 | DOCK1 |  |  | 1252 | 0.073 | 0.5313 | No |
| 16 | APBB2 |  |  | 1358 | 0.068 | 0.5258 | No |
| 17 | GNG5 |  |  | 1709 | 0.055 | 0.4787 | No |
| 18 | ARAP2 |  |  | 1845 | 0.051 | 0.4656 | No |
| 19 | LIFR |  |  | 1972 | 0.048 | 0.4533 | No |
| 20 | SEL1L3 |  |  | 2171 | 0.042 | 0.4285 | No |
| 21 | CDK6 |  |  | 2322 | 0.039 | 0.4109 | No |
| 22 | RHOJ |  |  | 2605 | 0.033 | 0.3711 | No |
| 23 | IDI1 |  |  | 2749 | 0.030 | 0.3530 | No |
| 24 | MGST3 |  |  | 2993 | 0.025 | 0.3182 | No |
| 25 | IL6ST |  |  | 3218 | 0.021 | 0.2856 | No |
| 26 | ITM2C |  |  | 3587 | 0.009 | 0.2281 | No |
| 27 | TMEM132B |  |  | 3600 | 0.009 | 0.2276 | No |
| 28 | LGALS3BP |  |  | 3757 | 0.003 | 0.2030 | No |
| 29 | RAB3GAP2 |  |  | 3789 | 0.001 | 0.1982 | No |
| 30 | FKBP9 |  |  | 4273 | -0.027 | 0.1252 | No |
| 31 | BMP7 |  |  | 4727 | -0.062 | 0.0627 | No |
| 32 | CARHSP1 |  |  | 5107 | -0.092 | 0.0173 | No |
| 33 | FGFBP3 |  |  | 5233 | -0.104 | 0.0146 | No |
| 34 | TNC |  |  | 5359 | -0.114 | 0.0136 | No |
| 35 | CDCA7L |  |  | 5720 | -0.164 | -0.0168 | No |
| 36 | PTPRZ1 |  |  | 5811 | -0.186 | -0.0002 | No |
| 37 | BRCA1 |  |  | 6197 | -0.435 | 0.0106 | No |
Table: GSEA details [plain text format]

  

Fig 2: POLLEN\_OUTERRADIALGLIA\_PMID\_26406371: Random ES distribution      
 Gene set null distribution of ES for **POLLEN\_OUTERRADIALGLIA\_PMID\_26406371**
